# Effect of La and Ce Microalloying on the Corrosion Resistance of 0.4Sb Low-Alloy Steel in a Harsh Marine Atmospheric Environment

**DOI:** 10.3390/ma19122685

**Published:** 2026-06-22

**Authors:** Qing Li, Xinyu Wang, Guowei Yang, Da Wei, Junjie Chen, Zhigao Wang, Jun Wang, Xiaojia Yang, Kui Xiao, Xiaogang Li, Zhong Li

**Affiliations:** 1 Key Laboratory for Corrosion and Protection (MOE), National Materials Corrosion and Protection Data Center, Institute for Advanced Materials and Technology, University of Science and Technology Beijing, Beijing 100083, China; 18030340508@163.com (Q.L.); wangjun@cei1958.com (J.W.);; 2 State Grid Sichuan Electric Power Research Institute, Chengdu 610041, China; jjchen1016@163.com (J.C.); wzg33@163.com (Z.W.); 3 Sichuan Chengdu Soil Environmental Material Corrosion National Observation and Research Station, Chengdu 610041, China; 4 State Key Laboratory of Environmental Adaptability for Industrial Products, China National Electric Apparatus Research Institute Co., Ltd., Guangzhou 510663, China

**Keywords:** La and Ce, marine atmosphere, corrosion mechanism

## Abstract

In this study, low-alloy structural steels with different La and Ce contents were prepared via vacuum smelting and controlled rolling and controlled cooling technologies, and their microstructures were characterized. The influence of La and Ce on the corrosion resistance of low-alloy steels was compared through indoor cyclic-immersion accelerated tests simulating tropical marine atmospheres. The corrosion mechanism of low-alloy steels with different La and Ce contents in simulated tropical marine atmospheres was investigated using electrochemical measurements and corrosion product analysis. The results show that La and Ce improve the uniform corrosion resistance of low-alloy steels. With increasing La/Ce content, the corrosion current density decreased from 1.8936 × 10^−6^ A cm^−2^ for 0LaCe to 1.29 × 10^−6^ A cm^−2^ for 0.3LaCe, corresponding to a reduction of approximately 31.9%. This is attributed to the fact that La/Ce addition promotes rust layer stabilization and densification, as suggested by the evolution of major rust phases and the presence of La/Ce-related oxidized species. Meanwhile, alloying with La and Ce improves the cracking of the rust layer, reduces the number of pores, and stabilizes the rust layer structure.

## 1. Introduction

It is well known that low-alloy steel is the most widely used structural material in the field of marine engineering [1]. However, low-alloy steel often suffers from severe corrosion failure, which, in mild cases, leads to material damage and a reduction in equipment service life, and, in severe cases, can trigger catastrophic accidents such as bridge collapses and pipeline explosions [2,3,4]. Improving the corrosion resistance of low-carbon and low-alloy steels and extending the service life of metallic equipment and components have long been major research focuses [5,6]. Generally, microalloying is the most common method for enhancing the corrosion resistance of low-alloy steel [5]. By adding trace alloying elements such as Cr, Cu, Ni, P, and Mo during steel smelting, the corrosion resistance of the substrate and the stability of the rust layer can be significantly improved, thereby reducing the corrosion rate of low-alloy steel [7,8,9,10,11,12,13]. Based on this principle, relatively mature weathering steels have been developed in China [14].

Rare earth elements (REEs) possess unique properties, and their excellent optical, magnetic, electrical, acoustic, and thermal properties can be utilized to develop new materials and devices with outstanding functional characteristics, earning them a reputation which has led to them being referred to as “industrial monosodium glutamate” [15]. Research over recent decades has revealed that REEs can play roles in purifying molten steel, modifying inclusions, refining microstructures, and microalloying [16,17,18]. Furthermore, the addition of trace REEs can not only improve the mechanical properties of materials, such as strength and impact toughness, but can also enhance their corrosion resistance [19,20,21]. Therefore, the application of REEs in low-alloy steel has attracted increasing attention [22,23,24,25]. China ranks first in the world in terms of rare earth resource reserves and production volume [15]. It is of great significance to leverage China’s rare earth resource advantages, fully leverage the special functions of REEs, and develop high-performance steel materials [26]. Thus, alloying with REEs represents an important direction for the design of novel high-strength, corrosion-resistant steels in the future, enabling materials to exhibit both excellent mechanical properties and corrosion resistance and to play a greater role in engineering applications [18,27].

In this study, low-alloy structural steels with different La and Ce contents were fabricated via vacuum melting and controlled rolling and cooling techniques [14,18,23]. A laboratory cyclic immersion accelerated test was employed to simulate the marine atmospheric environment with high temperature, high humidity, and high salt spray, and the influence of La and Ce contents on the corrosion resistance of low-alloy steel was clarified [14,22,23,24]. Combined with electrochemical measurements, rust layer characterization, and local corrosion analysis, the role of La/Ce addition in the corrosion evolution process was investigated. Finally, the effects of La and Ce on the microstructure of low-alloy steel, as well as its corrosion behavior and mechanism in a simulated marine atmospheric environment, were revealed through microstructural characterization, electrochemical measurements, phase analysis of corrosion products, localized corrosion analysis, and other methods [24,25].

## 2. Experimental Methods and Materials

### 2.1. Materials

Three low-alloy steels with different La and Ce contents were fabricated via vacuum melting and designated 0RE, 0.03LaCe, and 0.3LaCe, respectively. Table 1 presents the chemical compositions of the three low-alloy steels with different La and Ce contents, as determined by chemical analysis. Chemical compositions were determined by inductively coupled plasma optical emission spectrometry (ICP-OES); the measurement uncertainty for each element is within +/−5% of the reported value.

To ensure a predominantly bainitic microstructure, the steel billets were heat-treated using controlled rolling and cooling technology. First, the billets were heated to 1200 °C and held for 2 h to achieve uniform automatization, followed by furnace cooling to 1000 °C, after which the billets were removed for rolling. The initial rolling temperature was approximately 1000 °C, and the billets were rolled in 6 passes to obtain steel plates approximately 14 mm thick, with a finish rolling temperature of approximately 880 °C. Subsequently, the plates were rapidly cooled to around 440 °C by water penetration cooling, and finally air-cooled to room temperature.

### 2.2. Microstructure Characterization

Samples with dimensions of 10 mm × 10 mm × 3 mm were cut from the normal plane of the rolled steel plate using wire electrical discharge machining (WEDM) for microstructural observation. One 10 mm × 10 mm surface of each sample was designated as the working surface, which was sequentially ground with SiC sandpapers up to 2000 grit, followed by water-based polishing with 1.5 μm and 0.5 μm diamond pastes to achieve a mirror finish. The polished surface was then rinsed with deionized water and ethanol, respectively, and dried for subsequent characterization.

The polished specimens were examined under a field-emission scanning electron microscope (FEI Quanta 250, FEI Company, Hillsboro, OR, USA) to observe the morphology of typical inclusions, and energy-dispersive X-ray spectroscopy (EDS; X-Max detector, Oxford Instruments NanoAnalysis, High Wycombe, UK, operated in automatic mode with a standard element list including Fe, C, Mn, Si, Cr, Ni, Cu, Sb, Sn, La, and Ce) was used to determine the elemental distribution. For microstructural analysis, the polished working surface was etched with 4 vol.% nital (nitric acid in ethanol) for 10 s, immediately rinsed with deionized water and ethanol, and dried. The microstructure was then characterized using a stereomicroscope and SEM.

Electron backscatter diffraction (EBSD) was employed to analyze the crystallographic information of the steel. Specimens for EBSD observation were electropolished after mechanical polishing using an electrolyte composed of 10 vol.% perchloric acid and 90 vol.% ethanol, with a polishing voltage of 25 V and a duration of 10 s. EBSD data were acquired using TEAM data-collection software (version 4.5, EDAX Inc., Mahwah, NJ, USA) integrated with the SEM, with a scanning step size of 0.3 μm and an accelerating voltage of 30 kV. The corresponding EBSD data were subsequently analyzed using OIM 7.3.

### 2.3. Electrochemical Behavior Method

Electrochemical measurements were performed using a conventional three-electrode system, with the tested steel serving as the working electrode (10 mm × 10 mm × 3 mm), a platinum sheet as the counter electrode, and a saturated calomel electrode (SCE) as the reference electrode. A copper wire was spot-welded to the back of the working electrode, which was then encapsulated with epoxy resin, leaving an exposed working area of 1 cm^2^. The working surface was ground to at least 2000 grit with SiC sandpaper, rinsed with deionized water and ethanol, and quickly dried before testing. The electrolyte used for electrochemical measurements was a simulated marine atmospheric solution, consisting of 0.1 wt.% NaCl + 0.05 wt.% CaCl_2_ + 0.05 wt.% Na_2_SO_4_.

All electrochemical tests were conducted on a Princeton PARSTAT 3F electrochemical workstation (AMETEK Scientific Instruments, Oak Ridge, TN, USA). The specimen was first subjected to an open-circuit potential (OCP) measurement to ensure stabilization of the three-electrode system. The system was considered stable when the potential fluctuation of the sample was within ±20 mV over a 10 min period in the test solution, after which subsequent electrochemical impedance spectroscopy (EIS) and polarization measurements could be performed. The frequency range for EIS was set from 100 kHz to 10 mHz with an alternating current (AC) sinusoidal amplitude of 10 mV, and the obtained data were analyzed using ZsimpWin 3.5 software. For the potentiodynamic polarization test, the scan rate was 0.333 mV/s, and the potential scanning range was set at OCP ± 500 mV. The subsequent data fitting was completed using EClab software (version 11.10, Bio-Logic Science Instruments, Seyssinet-Pariset, France). All tests were carried out at room temperature. Each electrochemical test was repeated three times, and the fitting parameters are reported as averaged values with standard deviations.

### 2.4. Wet/Dry Cyclic Immersion Test

A cyclic immersion accelerated corrosion test was employed to simulate the wet–dry alternating marine atmospheric environment. Plate-shaped specimens with dimensions of 50 mm × 25 mm × 3 mm were cut, ground to 800 grit with SiC sandpaper, rinsed with deionized water and ethanol, and dried before being weighed and measured. The cyclic immersion accelerated test was conducted in a self-developed EA-08 cyclic immersion chamber, with a wet–dry cycle period of 30 min, including 7.5 min of immersion and 22.5 min of drying. The test temperature and relative humidity (RH) were controlled at 40 ± 1 °C and 90% RH, respectively. The test solution was an accelerated simulated marine atmospheric solution consisting of 5 wt.% NaCl + 0.05 wt.% CaCl_2_ + 0.05 wt.% Na_2_SO_4_. The test durations were set at 168 h, 336 h, 504 h, and 672 h. To improve test accuracy, three parallel specimens were prepared for each steel type at each test cycle.

After each test cycle, the specimens were removed for ultrasonic dedusting. The rust-removal solution was composed of 500 mL deionized water, 500 mL analytical-grade concentrated HCl (36–38 wt.%), and 3.5 g hexamethylenetetramine. The specimens were then rinsed thoroughly with deionized water and ethanol, dried, and subsequently weighed to calculate the corrosion rate. The formulas for calculating corrosion weight loss and corrosion rate are as follows:

(1) $$w\;=\;\frac{m_{0\;}-{\;m}_{t}}{S}$$

(2) $$v=\frac{{(m}_{0}-{\;m}_{t})\;\times \;10^{4}}{S\rho t}$$

where w is the corrosion weight loss (g/cm^2^), v is the corrosion rate (mm/y), m_0_ is the initial weight of the specimen (g), m_t_ is the weight of the specimen after rust removal (g), S is the exposed area of the specimen (cm^2^), ρ is the density of steel (7.8 g/cm^3^), and t is the exposure time (h).

### 2.5. Corrosion Product Analysis Method

The cross-sectional morphology of the rust layers on the tested steels was observed using scanning electron microscopy (SEM), and energy-dispersive X-ray spectroscopy (EDS) was employed to analyze the elemental distribution and content. The rust layers on the specimen surfaces after each test cycle were scraped off with a blade, and the rust particles were ground into powder using a quartz mortar for subsequent analysis of the rust composition.

X-ray diffraction (XRD) was used to analyze the phase composition of rust layers on Cr-containing steels after different cyclic immersion durations. The operating voltage and current were 40 kV and 150 mA, respectively, with a scanning rate of 2°/min and a diffraction angle range of 10–90°. Phase identification was performed by matching the observed diffraction peaks against standard JCPDS reference cards. Quantitative Rietveld refinement was not performed; therefore, relative peak intensities are used for qualitative discussion of phase evolution only.

X-ray photoelectron spectroscopy (XPS) was used to analyze the surface chemical states of the main alloying elements in the rust layers. The test was performed at a power of 150 W, with an operating voltage of 14.8 kV and current of 1.6 A, using an Al target as the X-ray source and a beam spot diameter of 650 μm. All test peaks were calibrated to the C 1s standard peak (284.8 eV) and fitted using XPS-PEAK 4.1 software. A Shirley background was applied, and peaks were fitted using mixed Gaussian–Lorentzian profiles (GL ratio 20–30%). Binding energy references for peak assignment were taken from the NIST X-ray Photoelectron Spectroscopy Database. The XPS fitting results were used to identify the chemical states of the main elements and compare the relative spectral features of the rust layers.

The corrosion morphology after rust removal was characterized by three-dimensional (3D) morphology using a laser confocal microscope, and statistical analysis was performed on the parameters related to corrosion pits.

## 3. Results

### 3.1. Microstructure Characterization

**Figure 1:**
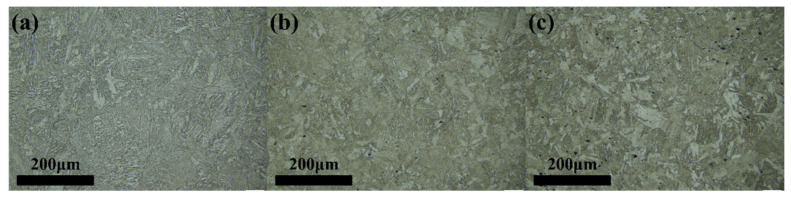
Microstructures of the three steels containing La and Ce: (**a**) 0RE, (**b**) 0.03LaCe, (**c**) 0.3LaCe.

**Figure 2:**
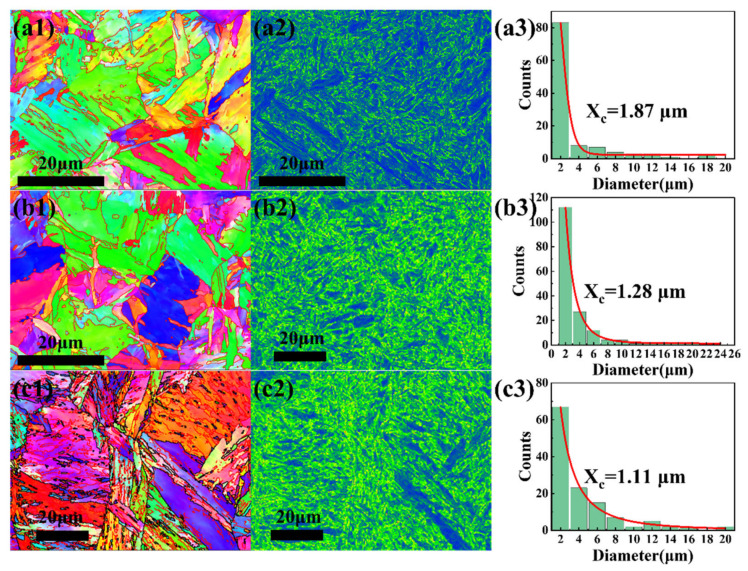
EBSD analysis of the three steels containing La and Ce: (**a1**–**a3**) 0RE, (**b1**–**b3**) 0.03LaCe, (**c1**–**c3**) 0.3LaCe; 1, 2, and 3 represent the IPF maps, KAM maps, and grain size statistical distribution maps, respectively.

Figure 1 shows the microstructural morphologies of the three steels containing La and Ce. It can be observed from the figure that the microstructures of all three steels are a mixture of lath bainite (LB, lath-like ferrite + retained austenite or martensite (MA)) and granular bainite (GB, equiaxed ferrite + island-like MA). However, the microstructural morphologies differ due to the varying La and Ce contents. As shown in Figure 2(a1), the 0RE steel is dominated by bainitic ferrite, with only a small amount of MA precipitated at the grain boundaries.

Rare earth oxides formed by rare earth elements possess a high melting point, which can remain solid during the solidification of the molten alloy and act as nucleation substrates. Meanwhile, they exhibit a low lattice misfit, reducing the nucleation energy and undercooling. Therefore, rare earth oxides such as Ce_2_O_3_ can serve as inoculants, greatly promoting nucleation and refining the microstructure during solidification. The figures show that the grain size decreases gradually with increasing La and Ce content.

Figure 2 presents the EBSD analysis results for the three steels containing La and Ce, with Figure 2(a1,b1,c1) showing the inverse pole figure (IPF) maps for each steel. None of the three steels exhibits a specific dominant crystal plane, and the overall crystallographic orientation is uniform. Furthermore, with the continuous increase in La and Ce contents in the steel, the number of subdrains increases and they become finer, and the intragranular orientation difference is significantly smaller than the intergranular orientation difference.

The average grain sizes of the three steels are shown in Figure 2(a3,b3,c3). The figures show that the average grain size of the steel decreases from approximately 1.87 μm to 1.11 μm with increasing La and Ce content, indicating that the addition of La and Ce can significantly refine the steel’s grain size. The kernel average misorientation (KAM) is commonly used to characterize the local lattice rotation of metallic materials and to qualitatively evaluate the distribution of local residual stress or local strain. As shown in Figure 2(a2,b2,c2), the KAM value increases slightly with the addition of La and Ce. The atomic radius of rare earth elements is much larger than that of Fe atoms, and their solid solubility in the ferrite lattice is extremely low. A large number of rare earth-rich inclusions are enriched at the grain boundaries, resulting in a higher KAM value at the interfaces.

### 3.2. Electrochemical Behavior

**Figure 3:**
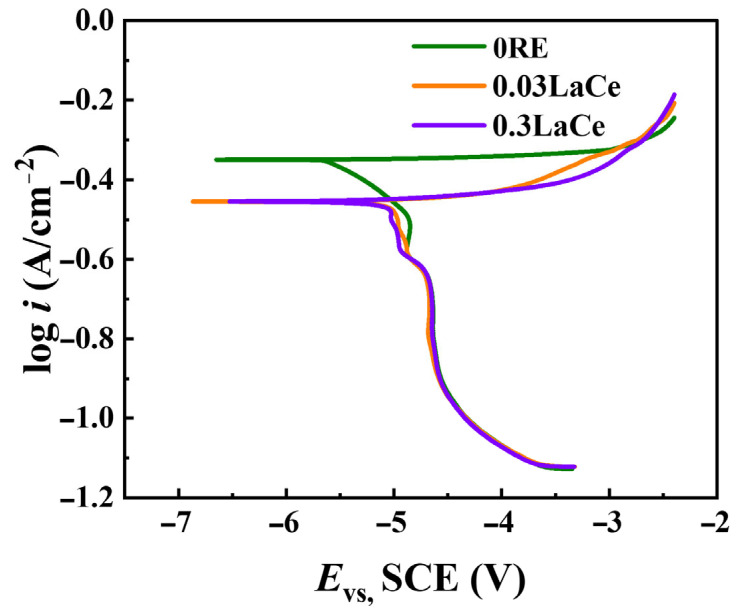
Potentiodynamic polarization curves of steels with different La and Ce contents in simulated marine atmospheric solution.

Figure 3 shows the potentiodynamic polarization curves of the three steels with different La and Ce contents in the simulated marine atmospheric solution. It can be observed that the curves exhibit a similar shape, indicating that the three steels share the same electrochemical mechanism: mixed control of anodic active dissolution, cathodic oxygen absorption, and hydrogen evolution reactions.

The addition of La and Ce in the steel leads to a leftward shift in the anodic polarization curves, and this effect is most pronounced when the La and Ce content reaches 0.3 wt.%, implying that the incorporation of La and Ce can suppress the active dissolution of the steel, with the 0.3LaCe steel showing the optimal inhibition effect. For the cathodic reaction, the curves shift significantly to the left upon addition of La and Ce, suggesting that these additives can also inhibit cathodic reactions on the steel surface.

The corrosion potential (*E*_corr_) and corrosion current density (*i*_corr_) of the three steels were obtained by fitting the polarization curves using the Tafel method, and the results are presented in Table 2. The addition of La and Ce causes a negative shift in the *E*_corr_ value. Owing to the high activity of rare earth elements, their addition increases the initial corrosion tendency of the low-alloy steel thermodynamically. However, the *i*_corr_ value of the low-alloy steel decreases substantially with the addition of La and Ce and continues to decline as the La and Ce content increases.

Overall, although La/Ce addition causes a negative shift in Ecorr, it decreases icorr from 1.8936 × 10^−6^ A cm^−2^ for 0RE to 1.29 × 10^−6^ A cm^−2^ for 0.3LaCe, indicating kinetic suppression of the corrosion reaction. For weathering steels, the protective ability of the rust layer is a more important indicator of corrosion rate than the rust layer itself. Therefore, the corrosion products of the three low-alloy steels with different La and Ce contents should be analyzed in subsequent work.

**Figure 4:**
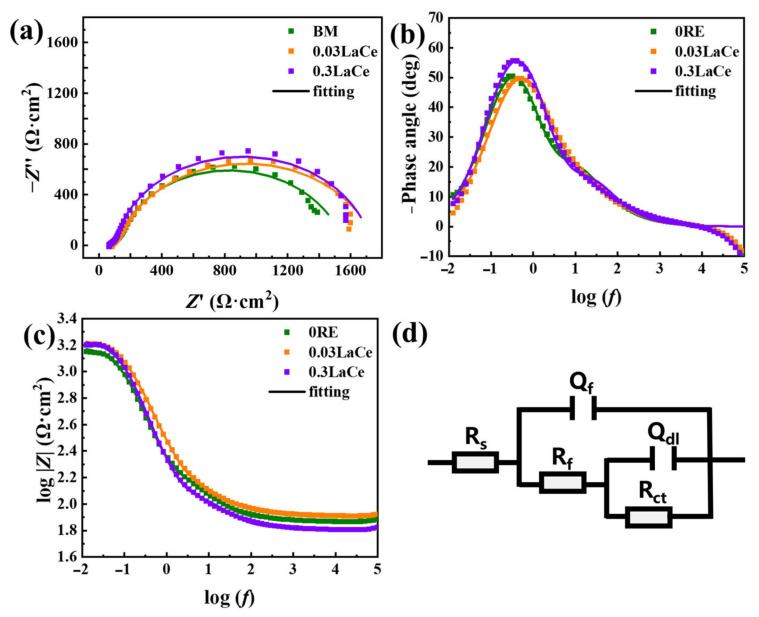
(**a**) Nyquist and (**b**) Bode plots, (**c**) Bode phase-angle plots, and (**d**) equivalent electrical circuit of steels with different La and Ce contents in simulated marine atmospheric solution of steels with different La and Ce contents in simulated marine atmospheric solution.

Figure 4 shows the electrochemical impedance spectroscopy (EIS) of the three steels containing La and Ce in the simulated marine atmospheric solution. As shown in the Nyquist plot (Figure 4a), the capacitive loops of the three steels exhibit a consistent shape, and their radii increase with increasing La and Ce content. This indicates that adding La and Ce can improve the corrosion resistance of steel in a marine atmosphere, and the effect becomes more significant with higher additions. As shown in Figure 4b, a characteristic peak appears in the phase angle plot at the low-frequency region (0.1–1 Hz). According to the literature, this peak corresponds to the anodic dissolution reaction on the steel surface. With increasing La and Ce content, the peak shifts to lower frequency and its intensity increases, suggesting improved corrosion resistance of the steel, consistent with the polarization curves.

To characterize the electrochemical behavior of the three steels more intuitively, the impedance spectra were fitted using the equivalent circuit shown in Figure 4d. It is worth noting that none of the three steels shows a complete semicircle in the Nyquist plot; instead, a semicircle rotation phenomenon is observed. This indicates that the “dispersion effect” occurs at the working electrode due to factors such as surface roughness and energy dissipation, resulting in a non-ideal capacitive response. Therefore, the constant phase element (Q) is used to represent the equivalent component. The equivalent circuit selected here has two time constants, where R_s_ is the solution resistance, *Q*_f_ and *R*_f_ are the constant phase element and the resistance corresponding to the corrosion product film, respectively, and *Q*_dl_ and *R*_ct_ are the constant phase element of the double layer and the charge transfer resistance on the steel surface, respectively. As shown in Figure 4, the fitted curves agree well with the measured data, demonstrating the rationality of the selected equivalent circuit.

Table 3 presents the fitting results of the parameters related to the impedance spectra. From the table, it can be observed that as La and Ce content in the steel increase, *R*_ct_ gradually increases and *Q*_dl_ gradually decreases. Table 3 presents the fitted EIS parameters. The averaged fitting results show that *R_ct_* generally increases and *Q_dl_* decreases with increasing La/Ce content. La and Ce additions increase the charge-transfer resistance from 1352 ± 67 Ω cm^2^ for 0RE to 1600 ± 80 Ω cm^2^ for 0.3LaCe, further supporting the kinetic inhibition of interfacial corrosion reactions.

### 3.3. Corrosion Rate

**Figure 5:**
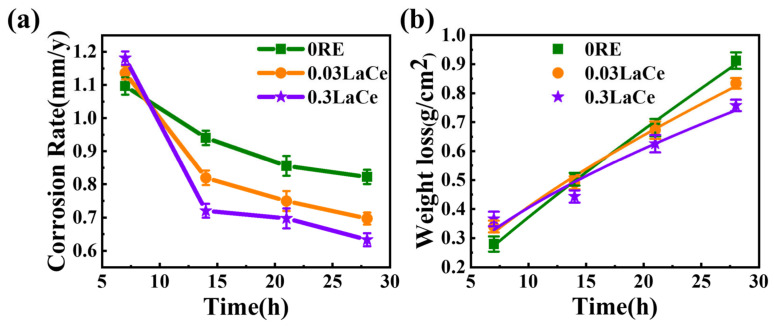
Corrosion kinetic curves of steels with different La and Ce contents in simulated marine atmospheric solution: (**a**) corrosion weight loss, (**b**) corrosion rate.

Figure 5 shows the corrosion weight loss, corrosion rate, and their fitting curves for the three steels containing La and Ce after the accelerated cyclic immersion test. It can be observed that the data of corrosion weight loss and corrosion rate both conform to the power exponential function (*V* = *At*^n^), and the fitting results of corrosion weight loss are listed in Table 4. According to the literature, this function has been widely used to evaluate the corrosion behavior of low-alloy steels, and its reliability has been verified by many researchers.

Figure 5a presents the corrosion rates of steels with different La and Ce contents after different cyclic immersion durations. The corrosion rates of the three low-alloy steels gradually decrease over time, and the corrosion products formed on their surfaces exhibit good protective properties. With the addition of La and Ce, the corrosion rate gradually decreases over time, indicating that the rust layer gradually acquires a protective effect on the steel matrix, thereby reducing the matrix’s corrosion rate. The above results demonstrate that the addition of La and Ce can effectively improve the corrosion resistance of steel in harsh atmospheric environments, and that as the total La + Ce content increased from 0.036 to 0.33 wt.%, the improvement in corrosion resistance became more pronounced. Meanwhile, it is noted that the corrosion rates of 0.03LaCe and 0.3LaCe are higher than that of 0RE at the initial stage of immersion, which is consistent with the results of the potentiodynamic polarization curves in the previous section. The addition of rare earth elements enhances the surface activity of the low-alloy steel, thereby increasing the initial corrosion rate. Only after a stable corrosion product layer forms on the surface of the low-alloy steel can the rust layer serve to block environmental media and delay further corrosion of the matrix.

As shown in Figure 5b, adding La and Ce to the steel significantly reduces corrosion weight loss, and this effect becomes more pronounced with increasing La and Ce content. The literature indicates that the exponent *n* in the power function can be used as an indicator of the physical and chemical properties of the rust layer; the lower the *n* value, the more protective the rust layer is for the steel. As shown in Table 4, with the gradual addition of La and Ce, the *n* value decreases to 0.5, indicating that a relatively protective rust layer forms on the steel surface with high La and Ce contents.

### 3.4. Corrosion Product Analysis

**Figure 6:**
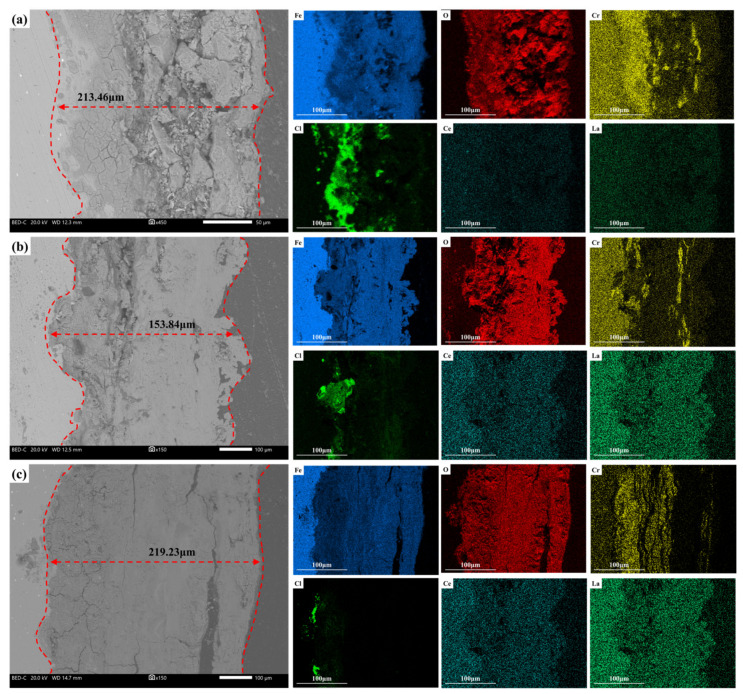
Cross-sectional morphology and elemental distribution of rust layers on three steels after 28 days of cyclic immersion accelerated testing: (**a**) 0RE, (**b**) 0.03LaCe, and (**c**) 0.3LaCe.

After 28 days of cyclic immersion, the rust layer thicknesses of 0RE, 0.03LaCe, and 0.3LaCe were 213.46 ± 12.8, 153.84 ± 9.2, and 219.23 ± 13.1 μm, respectively, and the 0.3LaCe steel exhibited a more compact rust layer with fewer connected cracks and pores. The 0RE (Figure 6a) rust layer is loose and defect-rich, with many pores and wide cracks. The 0.03LaCe (Figure 6b) sample forms a thinner layer with fewer defects, but local discontinuities remain. The 0.3LaCe (Figure 6c) sample forms a thicker, more compact layer, with finer, less connected cracks and a more intact rust/steel interface. EDS mapping indicates that Fe and O are the major constituents in all rust layers. Cl enrichment is strongest in 0RE and extends toward the inner layer, is reduced in 0.03LaCe, and is mainly confined to the outer region in 0.3LaCe. Cr enrichment near the inner rust region is more continuous in 0.3LaCe, while La and Ce are detectably distributed in the LaCe-bearing rust layers. La/Ce addition promotes rust layer compactness, reduces defect connectivity and Cl^−^ penetration, and facilitates the evolution of the rust layer toward a more protective structure. Although the rust layer of 0.3LaCe is slightly thicker than that of 0RE after 28 days, the improved rust layer protectiveness should not be attributed to thickness alone. Instead, it is mainly related to its higher compactness, lower defect connectivity, more continuous rust/steel interface, and reduced inward Cl^−^ penetration.

**Figure 7:**
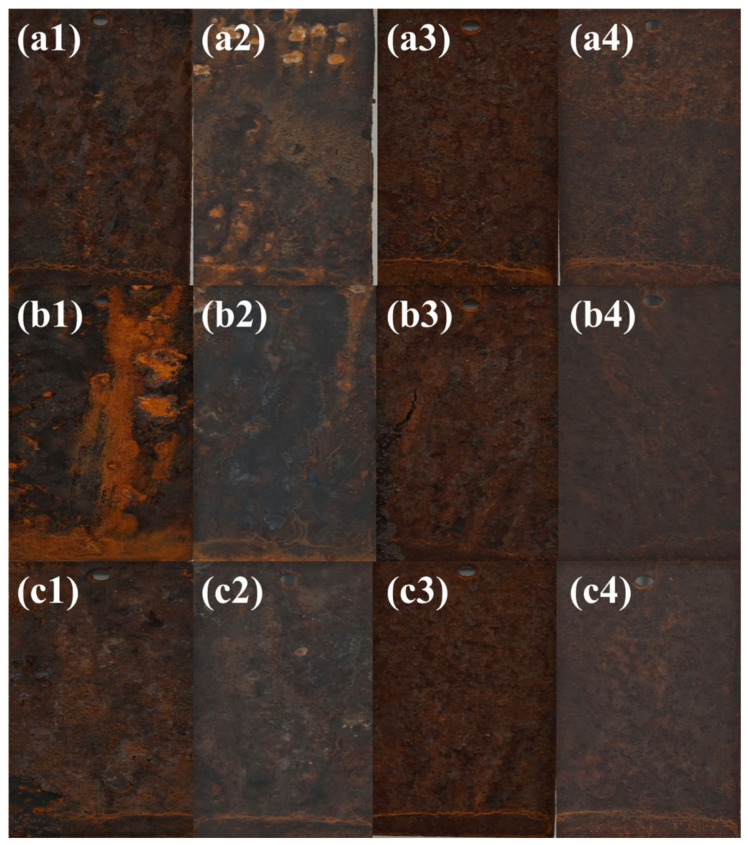
Macroscopic morphologies of surface corrosion products after cyclic immersion tests: (**a1**–**a4**) 0RE; (**b1**–**b4**) 0.03LaCe; (**c1**–**c4**) 0.3LaCe; 1, 2, 3, and 4 represent 168, 336, 504, and 672 h, respectively.

Figure 7 shows the macroscopic morphologies of corrosion products on the surface of low-alloy steels after different immersion periods. With increased immersion time, the color of corrosion products gradually deepens, reflecting the transformation of corrosion product types. The color evolution of corrosion products is generally associated with changes in the relative fractions of iron oxyhydroxides and oxides, such as γ-FeOOH, α-FeOOH, Fe_2_O_3_, and Fe_3_O_4_, as reported in previous studies [28].

**Figure 8:**
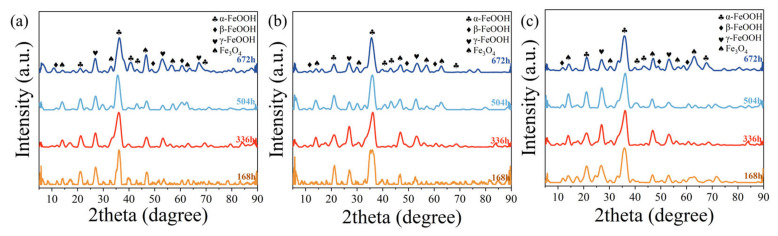
Phase composition of rust layers on steels with different La and Ce contents after cyclic immersion accelerated testing: (**a**) 0RE, (**b**) 0.03LaCe, and (**c**) 0.3LaCe.

After cyclic immersion accelerated testing, the rust layers on steels with different La and Ce contents were analyzed by XRD, as shown in Figure 8. XPS suggests the presence of La/Ce-related oxidized species in the rust layer. The figure shows that all rust layers had a similar phase composition, mainly Fe_3_O_4_, α-FeOOH, and γ-FeOOH. For 0RE, 0.03LaCe, and 0.3LaCe, the XRD peaks after 168, 336, 504, and 672 h were mainly assigned to α-FeOOH, β-FeOOH, γ-FeOOH, and Fe_3_O_4_. The phase types were basically the same at each time point, indicating that La and Ce additions did not change the main phase system of the rust layer. However, they affected the relative peak-intensity features and evolution of these corrosion products. With increasing corrosion time, the peaks became clearer, indicating improved crystallinity of the rust layers. Early active products gradually transformed into more stable products. Peak-intensity changes show that, at the later stage (504–672 h), the contribution of stable α-FeOOH increased, while the relative contribution of γ-FeOOH and β-FeOOH decreased. This indicates that the rust layer’s protective ability improved over time. Fe_3_O_4_ was detected at all stages, indicating that some conductive phase remained in the rust layer. A comparison at the same corrosion duration shows that La/Ce-containing steels, especially 0.3LaCe, exhibited a more obvious stabilization trend in the later stage. The mechanism can be explained as follows. During corrosion, La/Ce-related oxidized species may be enriched in the rust layer, as suggested by EDS mapping and XPS results. This process promotes the refinement and densification of the rust layer. It also reduces Cl^−^ transport and accumulation in the rust layer. As a result, the rust layer tends to evolve toward a denser and more protective structure with reduced Cl^−^ transport and defect connectivity.

**Figure 9:**
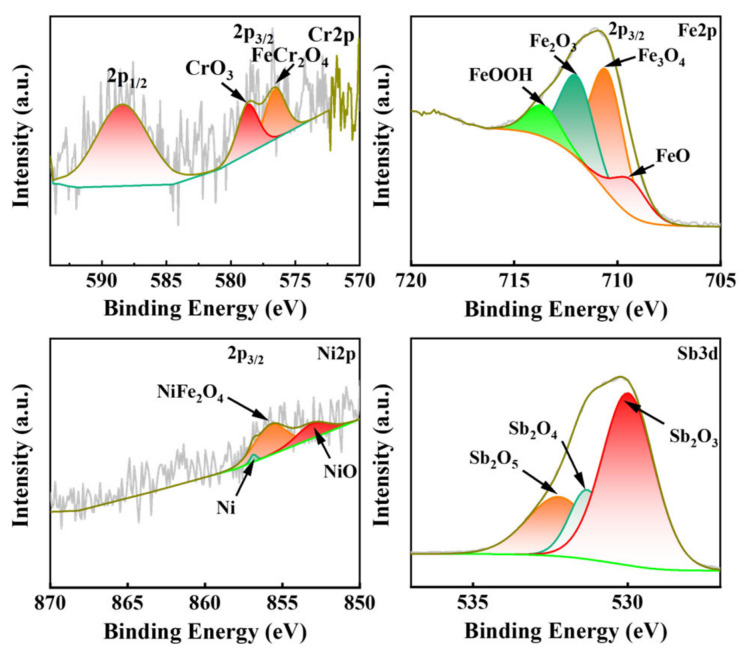
XPS analysis of the major alloying elements in the rust layer of 0RE steel after 672 h of cyclic immersion accelerated testing.

**Figure 10:**
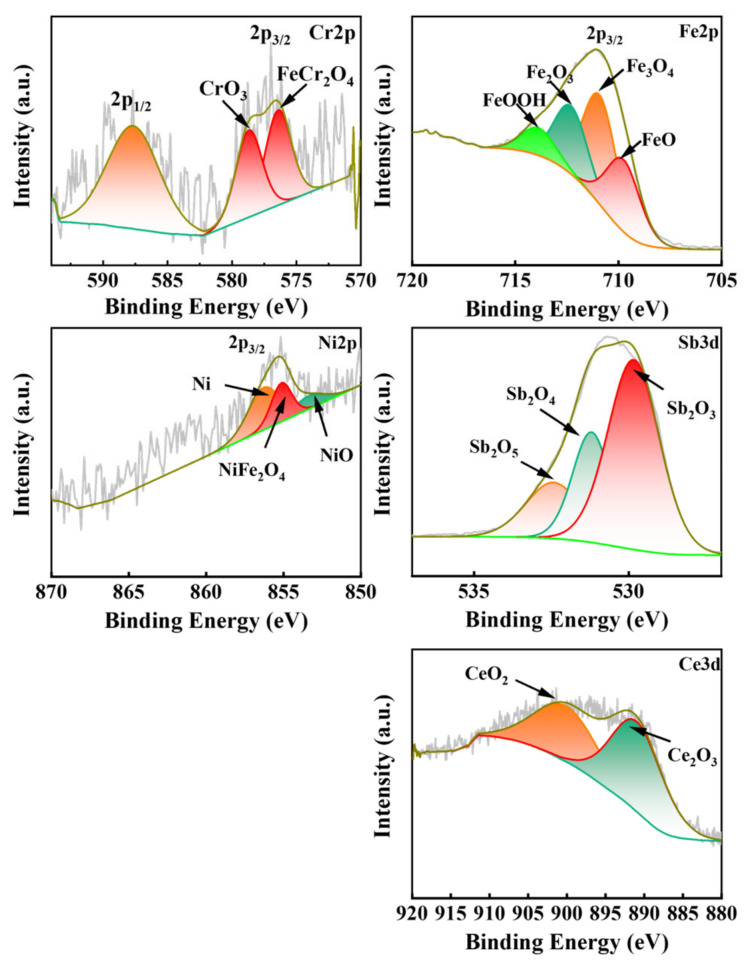
XPS analysis of the major alloying elements in the rust layer of 0.03LaCe steel after 672 h of cyclic immersion accelerated testing.

**Figure 11:**
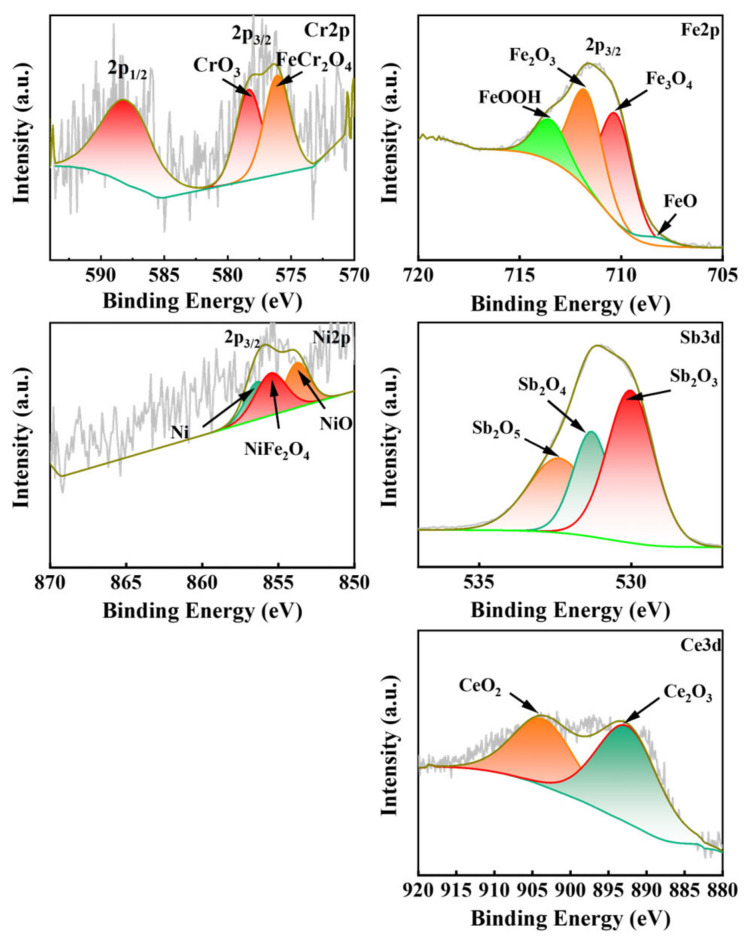
XPS analysis of the major alloying elements in the rust layer of 0.3LaCe steel after 672 h of cyclic immersion accelerated testing.

Obviously, the detection accuracy of XRD is insufficient to determine the existing states of alloying elements in the rust layer. To further clarify the composition of the rust layer, XPS analysis was performed on the main alloying elements in the rust layer, as shown in the Figure 9, Figure 10 and Figure 11. Fe exhibits four component peaks corresponding to Fe_3_O_4_, FeOOH, FeO, and Fe_2_O_3_, respectively. Cr exists in the forms of CrO_3_ and FeCr_2_O_4_, with relatively low peak intensities. For the 0.03LaCe and 0.3LaCe steels, the peak intensities of CrO_3_ and FeCr_2_O_4_ increase significantly, and such metal compounds play a beneficial role in the densification of the rust layer. For the Ce element, two substances, Ce(III)- and Ce(IV)-related oxidation states were observed, indicating that the addition of La and Ce is associated with the presence of rare earth-related oxidized species and a more stable rust layer structure [29]. This can also be seen from the XPS fitting results of Ni. After the addition of La and Ce, the types of compounds containing Ni remain unchanged, but the relative contents of NiFe_2_O_4_ and NiO increase, both of which contribute to improving the stability of the rust layer. The addition of La and Ce is associated with the presence of rare earth-related oxidised species and a more stable rust layer structure.

### 3.5. Local Corrosion Analysis

**Figure 12:**
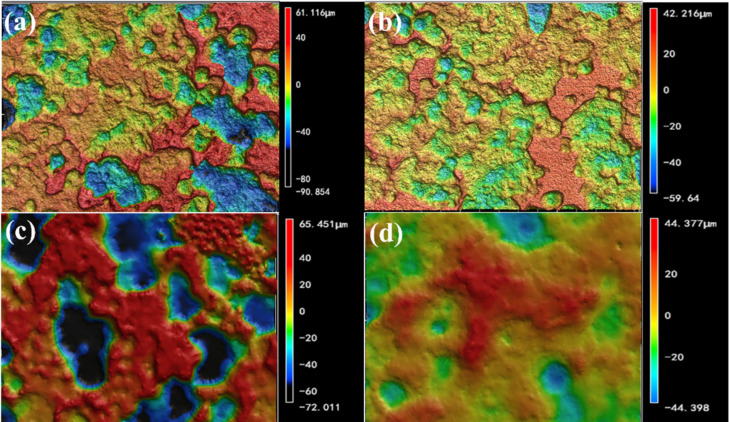
3D corrosion morphologies of 0RE steel after rust removal at different cyclic immersion periods: (**a**) 168 h, (**b**) 336 h (**c**) 504 h, (**d**) 672 h.

**Figure 13:**
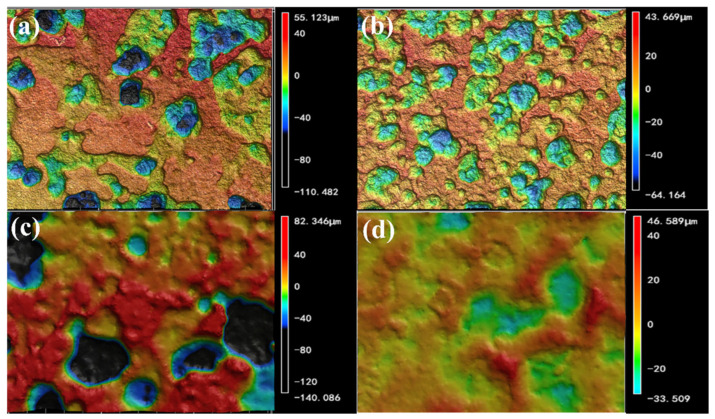
3D corrosion morphologies of 0.03LaCe steel after rust removal at different cyclic immersion periods: (**a**) 168 h, (**b**) 336 h (**c**) 504 h, (**d**) 672 h.

**Figure 14:**
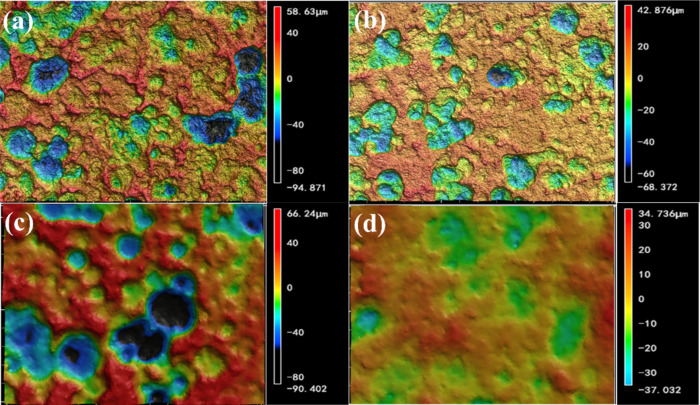
The 3D corrosion morphologies of 0.3LaCe steel after rust removal at different cyclic immersion periods: (**a**) 168 h, (**b**) 336 h (**c**) 504 h, (**d**) 672 h.

In the harsh marine atmospheric environment, in addition to excellent uniform corrosion resistance, the pit depth of low-alloy steels is also a critical indicator for their safe service. Therefore, laser confocal microscopy was employed to characterize the 3D morphologies of the three La- and Ce-containing steels after rust removal at different cyclic immersion periods, with a field of view of 4.5 mm × 6.5 mm; the number of pits identified and analyzed per field of view was as follows: 0RE: 284, 318, 347, and 382 at 168, 336, 504, and 672 h, respectively; 0.03LaCe: 213, 248, 271, and 296; 0.3LaCe: 187, 215, 238, and 262. The results are shown in Figure 12, Figure 13 and Figure 14.

Observation of the corrosion morphologies of the three steels reveals that at the initial corrosion stage, corrosion occurred over the entire field of view of the 0RE steel, with large and deep corrosion pits locally. In contrast, the corrosion degree of the 0.03LaCe and 0.3LaCe steels was relatively mild, and their surfaces showed numerous shallow local corrosion pits, particularly evident in the 0.3LaCe steel. This indicates that the steel’s inherent corrosion resistance increases with the addition of La and Ce, consistent with the electrochemical results in Figure 3.

With further extension of the cyclic immersion time, all three steels exhibited an overall uniform corrosion morphology at the later corrosion stage. However, it can be observed that the corrosion pits on the surfaces of the 0.03LaCe and 0.3LaCe steels had larger diameters and smaller depths.

**Figure 15:**
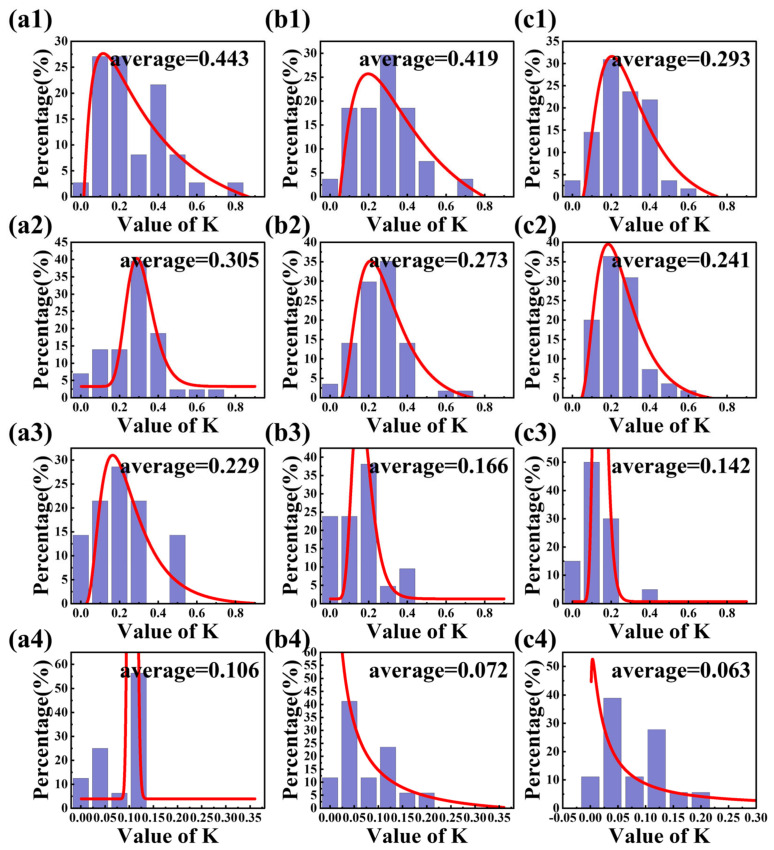
Statistics and distribution of K values for surface local corrosion pits at different cyclic immersion periods: (**a1**–**a4**) 0RE, (**b1**–**b4**) 0.03LaCe, (**c1**–**c4**) 0.3LaCe; 1, 2, 3, and 4 represent cyclic immersion for 168, 336, 504, and 672 h, respectively.

To more intuitively compare the morphological differences in the three steels after rust removal, the corrosion pits in the aforementioned field of view were statistically analyzed, and the parameter K and its distribution were used to characterize the pit features of the three steels, where K is the ratio of the maximum depth to the diameter of each pit. The results are shown in Figure 15.

Obviously, a larger K value indicates a more pronounced deep and narrow morphological characteristic of the pits, and a greater tendency for the pits to develop in depth. From the K value distribution diagrams, the K values of the three steels are mainly concentrated below 0.4, indicating that the pits are predominantly wide and shallow and that the three steels still mainly undergo uniform corrosion.

From the K value distribution diagrams of the three steels after 28 days of cyclic immersion, with increasing La and Ce contents, the average K value decreases from 0.106 ± 0.009 for 0RE to 0.085 ± 0.007 for 0.03LaCe and 0.063 ± 0.006 for 0.3LaCe. This fully demonstrates that the addition of La and Ce in steel inhibits the tendency for corrosion pits to develop in depth, causing local corrosion to tend toward a wide, shallow morphology, and this trend becomes more significant with higher contents. Therefore, La and Ce are clearly beneficial for suppressing local corrosion.

## 4. Conclusions

In this paper, three low-alloy steels with different La and Ce contents were prepared via vacuum smelting and controlled-rolling and controlled-cooling technologies. The mechanism by which La and Ce influence the microstructure of low-alloy steels and improve their corrosion resistance in a simulated marine atmosphere was analyzed. The results indicate that:

(1) The addition of La and Ce in steel exerts a significant influence on the electrochemical process of the steel. La/Ce addition causes a negative shift in corrosion potential, but decreases the corrosion current density and increases the charge-transfer resistance, indicating that the improvement in corrosion resistance is mainly associated with kinetic inhibition of the corrosion reaction. With increasing La/Ce content, the decrease in icorr and the increase in Rct become more evident, demonstrating enhanced resistance to active dissolution and interfacial charge transfer.
(2) La/Ce addition is associated with improved long-term corrosion resistance, which may be related to enhanced rust layer compactness, reduced defect connectivity, and restricted Cl^−^ penetration. Although the initial corrosion rate is slightly elevated due to the higher surface activity of rare earth additions, a progressively more protective rust layer forms over time, yielding lower long-term corrosion rates with increasing La/Ce content. The rust layer on La/Ce-containing steels shows improved protectiveness mainly through enhanced compactness, reduced defect connectivity, and restricted Cl^−^ penetration, rather than through rust layer thickening alone.
(3) The La- and Ce-containing oxidized species detected in the rust layer may contribute to modification of the originally loose and porous rust layer network by providing additional nucleation sites. This favors rust particle refinement and compact agglomeration, thereby reducing defects in the rust layer and hindering Cl^−^ penetration during long-term corrosion.
(4) The addition of La and Ce has a certain influence on the corrosion morphology of the steel. In the marine atmosphere, all three low-alloy steels still exhibit uniform corrosion. However, with increasing La and Ce content in the steel, the tendency for corrosion pits to develop in depth is progressively suppressed, as reflected by the decrease in average K value from 0.106 for 0RE to 0.063 for 0.3LaCe, indicating that La/Ce addition promotes a wider and shallower corrosion pit morphology.

## Figures and Tables

**Table 1:** Chemical compositions of the three tested materials (wt.%).

Sample	C	Si	Mn	P	S	Ni	Nb	Cu	Mo	Cr	Sb	La + Ce
0RE	0.05	0.50	1.50	≤0.005	≤0.008	1.0	0.05	0.5	0.25	2.0	0.42	-
0.03LaCe	0.05	0.50	1.50	≤0.005	≤0.008	1.0	0.05	0.5	0.25	2.0	0.40	0.036
0.3LaCe	0.05	0.50	1.50	≤0.005	≤0.008	1.0	0.05	0.5	0.25	2.0	0.40	0.33

**Table 2:** Tafel fitting results of potentiodynamic polarization curves.

Samples	E_corr_(mV)	I_corr_(×10^−6^ A/cm^2^)	β_a_	β_c_
0RE	−0.35353 ± 0.008	1.893 ± 0.096	8.4835	100.49
0.03LaCe	−0.45346 ± 0.011	1.829 ± 0.078	10.144	18.739
0.3LaCe	−0.45378 ± 0.009	1.290 ± 0.065	8.9279	11.585

**Table 3:** Impedance spectrum fitting results of steels with different La and Ce contents.

Sample	R_s_ (Ω·cm^2^)	Q_f_ (Ω^−1^·cm^−2^·s^n^)	R_f_ (Ω·cm^2^)	Q_dl_ (Ω^−1^·cm^−2^·s^n^)	R_ct_ (Ω·cm^2^)	χ^2^
0RE	75.16	5.425 × 10^−4^	137.7	7.358 × 10^−4^	1352± 67	5.35 × 10^−4^
0.03LaCe	82.86	3.323 × 10^−4^	88.2	5.246 × 10^−4^	1588± 74	1.81 × 10^−4^
0.3LaCe	65.44	3.472 × 10^−4^	69.7	7.419 × 10^−4^	1600± 80	1.38 × 10^−4^

**Table 4:** Fitting results of corrosion weight loss curves.

Equation	*V* = *At^n^*		
Steel	0RE	0.03LaCe	0.3LaCe
*A*	0.05145 ± 0.00671	0.08463 ± 0.01261	0.10468 ± 0.03149
*n*	0.85896 ± 0.04212	0.68301 ± 0.0488	0.58735 ± 0.09942
*R* ^2^	0.99677	0.98854	0.93302

## Data Availability

The original contributions presented in this study are included in the article. Further inquiries can be directed to the corresponding author.
